# Assessing risk of postoperative acute kidney injury in very elderly surgical patients using estimated glomerular filtration rate: we still need more researches

**DOI:** 10.1080/0886022X.2020.1754853

**Published:** 2020-04-20

**Authors:** Qin Wu, Hao Yang, Xi Zhong, Yan Kang

**Affiliations:** Department of Critical Care Medicine, West China Hospital, Sichuan University, Chengdu, China

We first thank Lei Wanet al. interests in our study [1,2]. In general, it is normally not accurate to assess the surgical burden using the scoring system like National Surgical Quality Improvement Program Risk (NSQIP) for serious complications, NSQIP for surgical risk calculator and so on retrospectively. Some key factors which involved in these scores are missing in our database and those data could not be obtained due to no well-organized follow-up of those patients who received surgery almost two years ago. The same goes for the postoperative risk factors. In order to avoid potential confounders, we only use factors we could obtain accurately in our study. For example, we compared the types of surgery, which we think is the most suitable for our research instead of any surgical burden score system. Without prospectively study design, it’s difficult to take all important aspects into account due to the data available and we believe this is an inevitable problem in all retrospective research. Indeed, this is one of the main limitations of our study. We encourage other authors to do more research in this area using a prospective study design.

We agree with Lei Wan et al. that postoperative AKI is not a single disease, but rather a syndrome comprising multiple clinical conditions. However, the main aim of our study is to assess risk of postoperative AKI, not the reasons of AKI. It would be better to rule out all the reasons of postoperative AKI. But it is always difficult to attribute the cause of postoperative AKI to a single factor [3]. Taking sepsis as an example, it is reasonable the onset of postoperative AKI may due to at least ischemia-reperfusion injury, infection, and nephrotoxic drug exposure [4]. We doubt if it is a better approach or not by adopting the way to investigate postoperative risk factors mentioned in Lei Wan et al. letter. We instead included the most important factors which may be related to postoperative AKI we could obtain accurately in our study. We believe that it is more meaningful to use less, but accurate data instead of more but inaccurate data for analysis.

In the multivariable model, due to the limited size of study population, a maximum 3 risk factors could be selected in the model in order to avoid overfitting of the model (at least 10–15 observations for each risk factor in a regression model). It is a way to do the sensitivity analysis, as shown in Table 5 of our study [1].

In summary, postoperative AKI is a topic of great concern to all. The highlights of our study are that this is the first study to validate the role of eGFRpreSurg in predicting postsurgical AKI among very elderly patients. Due to the design of the study and the quality of the data, we can’t come to the final conclusion in this field. Again, we express our sincere thanks to Lei Wan et al. interests in our study. And We look forward to more studies in the future.

## References

[1] Wu Q, Yang H, Bo H, et al. Predictive role of estimated glomerular filtration rate prior to surgery in postsurgical acute kidney injury among very elderly patients: a retrospective cohort study. Ren Fail. 2019;41(1):866–874. 31517563 10.1080/0886022X.2019.1662440 PMC6758700

[2] Wan L, Xue F-S, Liu S-H, et al. Assessing association of preoperative estimated glomerular filtration rate with postoperative acute kidney injury in very elderly surgical patients. Ren Fail. 2020. 10.1080/0886022X.2020.1760886 PMC7241471 32364042

[3] Goren O, Matot I. Perioperative acute kidney injury. Br J Anaesth. 2015;115(Suppl 2):ii3–14. 26658199 10.1093/bja/aev380

[4] Gomez H, Kellum JA. Sepsis-induced acute kidney injury. Curr Opin Crit Care. 2016;22(6):546–553. 27661757 10.1097/MCC.0000000000000356 PMC5654474

